# Vamorolone for Duchenne Muscular Dystrophy: A Cross-Trial Efficacy Comparison With Classic Corticosteroids From the FOR-DMD Trial

**DOI:** 10.1212/WNL.0000000000214756

**Published:** 2026-05-27

**Authors:** Paula R. Clemens, Anders Berglund, Marianela Schiava, Meredith K. James, Michael P. McDermott, Katherine Bushby, Erik Lampa, Edward Thomas James Rochford, Leanne M. Ward, Robert C. Griggs, Eric P. Hoffman, Michela Guglieri

**Affiliations:** 1 Department of Neurology, University of Pittsburgh School of Medicine, PA;; 2 Department of Veterans Affairs Medical Center, Pittsburgh, PA;; 3 Epistat AB, Uppsala, Sweden;; 4 John Walton Muscular Dystrophy Research Centre, Clinical and Translational Research Institute, Newcastle University and Newcastle Hospitals NHS Foundation Trust, United Kingdom;; 5 Department of Biostatistics and Computational Biology, University of Rochester Medical Centre, NY;; 6 John Walton Muscular Dystrophy Research Centre, Clinical and Translational Research Institute, Newcastle University, United Kingdom;; 7 Freelance Medical Writer, Italy;; 8 Department of Pediatrics, University of Ottawa, Division of Endocrinology and Metabolism, Children's Hospital of Eastern Ontario, Canada;; 9 Department of Neurology, University of Rochester Medical Centre, NY; and; 10 Department of Pharmaceutical Sciences, School of Pharmacy and Pharmaceutical Sciences, Binghamton University-SUNY, NY.

## Abstract

**Background and Objectives:**

Vamorolone demonstrated similar efficacy for Duchenne muscular dystrophy (DMD) compared with prednisone in a 24-week exploratory analysis and may reduce key side effects compared with classic corticosteroids. In this study, we compare the efficacy and anthropometric effect of vamorolone 6 mg/kg/d with prednisone 0.75 mg/kg/d and deflazacort 0.9 mg/kg/d in steroid-naïve boys aged 4 to <7 years using data from 2 trials.

**Methods:**

VISION-DMD was a phase 2b, 48-week randomized, double-blind trial assessing vamorolone 2 and 6 mg/kg/d vs placebo and prednisone 0.75 mg/kg/d to 24 weeks. Finding the Optimum Steroid Regimen for DMD (FOR-DMD) was a double-blind, parallel-group randomized trial comparing daily prednisone 0.75 mg/kg/d, daily deflazacort 0.9 mg/kg/d, and intermittent prednisone 0.75 mg/kg (10/10 days on/off). Entropy balancing generated weighted data for mixed model for repeated measures analyses that compared VISION-DMD vamorolone 6 mg/kg/d efficacy and anthropometric outcomes with FOR-DMD prednisone and deflazacort outcomes at 6 and 12 months. Inclusion criteria were boys with genetically confirmed DMD, age 4 to <7 years, ambulatory, and able to complete a time-to-stand (TTSTAND) assessment in <10 seconds at baseline.

**Results:**

All interventions showed motor improvements relative to baseline at 6 and 12 months. Using the weighted cohorts and at 12 months, vamorolone 6 mg/kg/d (n = 28) had similar changes in TTSTAND velocity, the primary motor endpoint component in both trials, vs daily prednisone (n = 50) or deflazacort (n = 55; mean baseline age 5.42 years all groups), but with CIs overlapping minimal clinically important thresholds of 0.023 rises per second (TTSTAND velocity least squares mean [LSM] difference [95% CI]: vamorolone 6 mg/kg/d vs prednisone 0.75 mg/kg/d, 0.004 rises per second [−0.025 to 0.032] and vs deflazacort 0.9 mg/kg/d, 0.001 rises per second [−0.027 to 0.028]). Body mass index (BMI) *Z*-scores increased in all groups, greatest with vamorolone (12-month LSM difference [95% CI]: vamorolone vs prednisone 0.57 [0.24–0.90]; vamorolone vs deflazacort 0.29 [0.03–0.56]), whereas growth slowdown occurred in prednisone- and deflazacort-treated boys but not vamorolone (12-month LSM difference in height *Z*-scores [95% CI]: vamorolone vs prednisone 0.57 [0.24–0.90]; vamorolone vs deflazacort 0.72 [0.53–0.91]).

**Discussion:**

Vamorolone demonstrated numerically similar TTSTAND velocity changes to prednisone and deflazacort at 1 year; however, interpretations of differences are limited by 95% CIs crossing minimally important difference thresholds. Further evidence of the growth-protective effect of vamorolone was observed; however, all treatments increased BMI. Vamorolone provides a linear growth-protective classic corticosteroid alternative.

**Trial Registration Information:**

NCT03439670; NCT01603407.

**Classification of Evidence:**

This Class III study did not definitively identify differences in efficacy between vamorolone and classic corticosteroids but found that vamorolone protects linear growth in boys with DMD.

## Introduction

Duchenne muscular dystrophy (DMD) is an X-linked progressive neuromuscular disorder caused by *DMD* gene variants that prevent dystrophin production, which affects approximately 1 in 5,000 male births.^1-4^ The lack of dystrophin leads to progressive limb function deterioration accompanied by cardiac and pulmonary involvement and death at a median age range of 21–40 years.^5,6^ Classic oral corticosteroids for DMD, prednisone and deflazacort, have established efficacy and can increase muscle strength and function over a certain period of time, delay loss of ambulation and upper-limb motor function after loss of ambulation, delay respiratory and cardiac function decline, and potentially increase life expectancy.^7-10^ As such, guidelines recommend the use of corticosteroids in patients with DMD^11^; however, long-term treatment is associated with side effects and safety concerns that can lead to low adherence to practice guidelines and decreased efficacy.^7,8^ As such, real-world dosing of classic corticosteroids is variable, with average prednisone dosages at 75% of the recommended starting dosage and deflazacort at 83% in ambulatory boys.^8,12^

The Finding the Optimum Steroid Regimen for DMD (FOR-DMD) trial (NCT01603407) was an international randomized, double-blind, parallel-group study that compared the long-term efficacy and adverse event profiles of the 3 most prescribed classic corticosteroid regimens over the initial 36 months of treatment in untreated boys with DMD.^13^ Although not placebo controlled, the FOR-DMD study showed daily prednisone 0.75 mg/kg/d and daily deflazacort 0.9 mg/kg/d, initiated in young corticosteroid-naïve boys with DMD, had similar efficacy through 36 months.^13^ All classic corticosteroid regimens were associated with motor function improvement; however, daily regimens were more effective than intermittent prednisone (10 days on/10 days off) in the composite primary efficacy outcome variable of the trial. Motor function outcomes, including time to stand from supine (TTSTAND) velocity, time to run/walk (TTRW) 10 meters velocity, 6-minute walk test (6MWT) distance in meters, and North Star Ambulatory Assessment (NSAA) total score, did not significantly differ between daily regimens. All treatments caused weight gain and slowdown in growth, with daily or intermittent prednisone associated with greater weight gain than daily deflazacort, whereas growth slowdown was greatest with daily regimens, most prominently deflazacort.^13^ Based on this balance of efficacy and safety, FOR-DMD supported initial daily dosing of prednisone or deflazacort over intermittent prednisone dosing for boys with DMD 4 years and older.

Vamorolone, a dissociative corticosteroid, was developed as an alternative to classic corticosteroids and is approved for DMD in several countries.^14^ Vamorolone is distinct from classic corticosteroids by 3 structure/activity relationships. First, vamorolone interaction with the glucocorticoid receptor differs leading to an increased binding of corepressors and decreased binding of coactivators, resulting in reduced glucocorticoid-response-element–mediated positive gene transcription while retaining anti-inflammatory nuclear factor-κB inhibition.^15,16^ Second, unlike prednisone and deflazacort, vamorolone exhibits mineralocorticoid receptor antagonist activity such as eplerenone.^17^ Third, vamorolone is not a substrate for 11β-hydroxysteroid dehydrogenase enzymes, which modulate prednisone and deflazacort prodrug to active-drug conversion.^17-19^

The pivotal vamorolone VISION-DMD trial (NCT03439670) was a randomized, double-blind, 24-week study, with placebo and prednisone control arms. After the initial 24 weeks, a further 24-week follow-up was conducted in which boys receiving vamorolone maintained their dosage and boys originally receiving prednisone or placebo switched to vamorolone after a 4-week washout.^20,21^ VISION-DMD demonstrated improvement in the primary outcome of change in TTSTAND velocity with vamorolone 6 mg/kg/d relative to placebo, and 4 ranked secondary motor outcomes.^21^ In an exploratory analysis, the efficacy of vamorolone 6 mg/kg/d was not significantly different from that of prednisone 0.75 mg/kg/d for all motor function outcomes over 24 weeks.^21^ After VISION-DMD, vamorolone was approved for DMD at a recommended starting dosage of 6 mg/kg/d, and the possibility to down-titrate to 4 or 2 mg/kg/d in response to side effects.^14^

In addition to the 24-week prednisone comparison in VISION-DMD, vamorolone data from 3 consecutive open-label clinical trials, totaling 30 months of treatment, including a 2–6 mg/kg/d group (67% of which was 6 mg/kg/d) were compared indirectly with classic corticosteroids using the CINRG Duchenne Natural History Study (CINRG DNHS) and North Star United Kingdom Network datasets, which included a mix of prednisone and deflazacort, daily and intermittent regimens.^22^ Treatment effects on TTSTAND velocity, time to climb 4 stairs (TTCLIMB) velocity, TTRW velocity, and NSAA total score through 30 months were not significantly different between vamorolone and combined historical corticosteroid regimens.^22^ Furthermore, in both VISION-DMD and open-label/pooled historical comparisons, vamorolone was associated with improved linear growth velocity and serum bone turnover markers compared with classic corticosteroids, including during the post-crossover period of VISION-DMD when participants switched from prednisone to vamorolone. In addition, there was descriptive evidence from VISION-DMD that vamorolone might reduce behavioral adverse event frequency vs prednisone.^20^

A recent systematic review described the efficacy of vamorolone over placebo and the possible adverse event reduction of vamorolone vs classic corticosteroids.^23^ However, VISION-DMD, a key component of this assessment, was designed for the primary TTSTAND velocity endpoint comparison of vamorolone with placebo at week 24 and not for week 48 analysis. Furthermore, VISION-DMD was not designed to maintain the Type I error probability for comparisons of each vamorolone group with prednisone. To better inform treatment decisions, there remains a need to compare the relative safety and efficacy of vamorolone with prednisone beyond the 24 weeks of VISION-DMD and with deflazacort beyond the pooled historical control that included a mixture of treatments and regimens. The VISION-DMD and FOR-DMD trials represent an opportunity to compare these treatments using randomized clinical trial data at the recommended starting dosages of the treatments. Therefore, the current analysis was performed to assess efficacy and anthropometric outcomes up to 1 year of treatment with vamorolone, prednisone, or deflazacort.

## Methods

### VISION-DMD Study Design and Methodology

VISION-DMD was a phase 2b, randomized, double-blind, placebo-controlled trial assessing the safety and efficacy of vamorolone at daily dosages of 2 mg/kg or 6 mg/kg as an oral suspension.^21^ The 24-week period 1 had 4 treatment groups (placebo, prednisone 0.75 mg/kg/d, vamorolone 2 mg/kg/d, vamorolone 6 mg/kg/d). After period 1, the placebo and prednisone groups crossed over to vamorolone (2 or 6 mg/kg/d, using baseline randomization) after a 4-week washout period, whereas the vamorolone groups continued uninterrupted on the same doses. The double blind was maintained throughout the 48-week study. The study was conducted at 33 sites in 11 countries between 2018 and 2021. Boys enrolled in VISION-DMD were corticosteroid-naïve, 4 to <7 years of age, able to perform TTSTAND unassisted in <10 seconds, and had genetically confirmed DMD. The primary efficacy outcome variable was TTSTAND velocity [rises per second] change from baseline to week 24 with the primary comparison being between vamorolone 6 mg/kg/d and placebo. Secondary outcome variables included 6MWT distance (in meters) and TTRW velocity (vamorolone 2 mg/kg/d and 6 mg/kg/d [in meters/second]) with secondary comparisons to placebo. TTCLIMB velocity and NSAA total score were exploratory outcomes. Secondary comparisons were performed between vamorolone 2 mg/kg/d and placebo. Most comparisons between the vamorolone doses and prednisone were exploratory, except 6MWT for which vamorolone/prednisone comparisons were listed as secondary.^20,21^

### FOR-DMD Study Design and Methodology

FOR-DMD was a randomized, double-blind, parallel-group trial that compared daily prednisone 0.75 mg/kg, daily deflazacort 0.90 mg/kg, and intermittent prednisone 0.75 mg/kg/d (10 days on/10 days off), administered as oral tablets.^13^ The study was conducted at 32 sites in 5 countries with enrollment between 2013 and 2016 and a 3-year assessment period. FOR-DMD participants were corticosteroid-naïve, 4 to <8 years of age, able to perform TTSTAND, and had genetically confirmed DMD. The composite primary outcome variable of the study was TTSTAND velocity (rises per second), forced vital capacity (in liters), and participant or parent global satisfaction measured by the Treatment Satisfaction Questionnaire for Medication, each averaged across all follow-up visits (baseline through month 36). Secondary efficacy outcome variables included TTRW velocity (in meters/second), NSAA total score, and 6MWT distance (in meters), with evaluations at screening, baseline, months 3 and 6, and every 6 months until month 36. The same prednisone dosing scheme was used in both FOR-DMD and VISION-DMD up to 39.9 kg of body weight.^13,21^

### Anthropometric Measurements

In both VISION-DMD and FOR-DMD, standing height was measured by a calibrated stadiometer, weight was measured using a calibrated scale, and body mass index (BMI) was calculated as weight/height^2^. Height, weight, and BMI *Z*-scores and percentiles were derived using male Centers for Disease Control and Prevention weight-/stature-/BMI-for-age charts representing 2–20 year olds.^24^

### Standard Protocol Approvals, Registrations, and Patient Consents

Both studies had written informed consent from the parent or legal guardian and assent from participants before initiation. Protocols were approved by the institutional review board or research ethics committee at each site. Studies were conducted in accordance with the International Conference on Harmonisation guidelines for Good Clinical Practice and the World Medical Association Declaration of Helsinki. Reporting results followed Consolidated Standards of Reporting Trials guidelines.

### Statistical Analyses

#### Study Cohorts and Balancing

All boys who received vamorolone in period 1 and ≥1 study medication dose during period 2 of VISION-DMD were considered for analysis. Boys from FOR-DMD were selected based on the key baseline inclusion criteria at baseline of genetically confirmed DMD, age 4 to <7 years, able to walk independently, and able to complete TTSTAND without assistance in <10 seconds. Entropy balancing was applied to baseline variables of TTSTAND velocity, age in years, NSAA total score, weight *Z*-score, and height *Z*-score between the treatment groups. Briefly, entropy balancing is a reweighting technique that assigns weights to boys in FOR-DMD treatment groups such that the mean values and standard deviations of the weighted covariate distributions align with those in the vamorolone-treated boys in VISION-DMD.^25^ An average treatment effect in the treated (ATT) weight was calculated and quality of balance assessed by comparing standardized mean differences (SMDs) and variance ratios for covariates before and after weighting. For the main analysis of this manuscript, vamorolone 6 mg/kg/d from VISION-DMD was balanced and compared with the prednisone 0.75 mg/kg/d and deflazacort 0.9 mg/kg/d groups from FOR-DMD. These comparisons were selected based on the recommended starting dosage of each of these treatments in the absence of additional factors. In addition, a separately weighted comparison between vamorolone 2 mg/kg/d from VISION-DMD and intermittent prednisone 0.75 mg/kg 10 days on/10 days off from FOR-DMD is summarized.

The 6- and 12-month efficacy and anthropometric changes from baseline in the entropy-balanced treatment groups were compared using the mixed model for repeated measures (MMRM), accommodating missing data under the missing-at-random assumption with weighted restricted maximum likelihood, using ATT weights. The MMRM used change from baseline as the outcome variable and adjusted for baseline value, baseline age, and an interaction between treatment and time point. Baseline values and age were modeled using restricted cubic splines with 3 internal knots placed at the 10th, 50th, and 90th percentile of each variable's marginal distribution. Pairwise comparisons using least squares mean (LSM) contrasts were made to assess differences between treatment groups. A heterogeneous autoregressive order 1 covariance structure was applied to each MMRM, and the denominator degrees of freedom was approximated using the Kenward-Roger method.^26^ There was no predefined order of the endpoints or method to account for multiple testing, and no power analysis was performed given that the sample sizes were already fixed. Statistical analyses were conducted in R version 4.4.2.

### Data Availability

Anonymized data not published within this article will be made available upon reasonable request from any qualified investigator. Specifically, the original VISION-DMD data may be provided after a Data Summary Request at the CINRG website (cinrgresearch.org/publications/data-summary-requests/) and after proposal approval with a signed data access agreement.

## Results

### Baseline Characteristics of Analysis Cohorts

**Figure 1:**
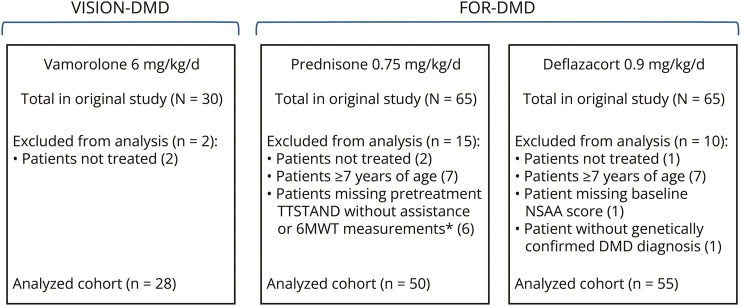
Analyzed Cohorts Used in the Cross-Study Comparisons of Vamorolone 6 mg/kg/d, Prednisone 0.75 mg/kg/d, and Deflazacort 0.9 mg/kg/d *Patients had baseline/screening TTSTAND values; however, these were excluded because of the requirement for assistance or incomplete tests. 6MWT = 6-minute walk test; d = day; DMD = Duchenne muscular dystrophy; TTSTAND = time-to-stand from supine; NSAA = North Star Ambulatory Assessment.

The entropy-balanced weighted groups consisted of 28 boys who received vamorolone 6 mg/kg/d, 50 boys who received daily prednisone 0.75 mg/kg/d, and 55 boys who received daily deflazacort 0.9 mg/kg/d (Figure 1). During VISION-DMD, 2 patients receiving vamorolone 6 mg/kg/d discontinued (adverse event and withdrawal of consent); they were included in the present analysis. In the FOR-DMD prednisone group, 6 patients were excluded for not completing the baseline TTSTAND assessment fully without assistance, despite having TTSTAND values recorded. A single subject in the FOR-DMD deflazacort group was excluded for not having genetically confirmed DMD (a protocol waiver was granted during the original study based on the absence of dystrophin observed in the muscle biopsy).

The entropy-balanced baseline characteristics of the weighted treatment groups with SMD values are shown in Table 1. The estimated SMD for each of the baseline weighting variables confirmed the suitability of the matching approach (eFigures 1 and 2). Boys from FOR-DMD had longer exposure to treatment than the VISION-DMD vamorolone-treated boys with a wider range of values driven by a minority of premature dropouts and boys who had late month 12 visits. Baseline TTSTAND velocity and NSAA total scores were well matched by the weighting approach; however, a degree of variation occurred in the baseline 6MWT distance and TTRW velocity in the weighted data. Boys in the prednisone group had higher mean baseline 6MWT distance and lower mean TTRW velocity values than boys in the deflazacort and vamorolone 6 mg/kg/d groups. In particular, the variation in baseline 6MWT distance resulted in SMD values greater than ±0.5 for the prednisone/vamorolone and prednisone/deflazacort comparisons.

**Table 1:** Baseline Characteristics of Weighted Treatment Cohorts and Standardized Mean Difference Measures of Balancing

	Vamorolone 6 mg/kg/d VISION-DMD (n = 28)	Prednisone 0.75 mg/kg/d FOR-DMD (n = 50)	Deflazacort 0.9 mg/kg/d FOR-DMD (n = 55)	SMD: Vamorolone 6 mg/kg/d vs prednisone 0.75 mg/kg/d	SMD: Vamorolone 6 mg/kg/d vs deflazacort 0.9 mg/kg/d	SMD: Prednisone 0.75 mg/kg/d vs deflazacort 0.9 mg/kg/d
Age, y, mean ± SD	5.42 ± 0.88	5.42 ± 0.87	5.42 ± 0.87	0.00	0.00	0.00
Height, cm, mean ± SD	107 ± 7	108 ± 6	108 ± 8	−0.15	−0.08	−0.06
Height *Z*-score, mean ± SD	−1.04 ± 1.05	−1.04 ± 1.04	−1.04 ± 1.04	0.00	0.00	0.00
Weight, kg, mean ± SD	19.06 ± 2.94	19.25 ± 2.11	19.40 ± 3.93	−0.07	−0.10	0.04
Weight *Z*-score, mean ± SD	−0.32 ± 1.02	−0.32 ± 1.01	−0.32 ± 1.01	0.00	0.00	0.00
BMI, kg/m^2^, mean ± SD	16.57 ± 1.45	16.53 ± 1.44	16.62 ± 1.60	0.03	−0.03	0.06
BMI *Z*-score, mean ± SD	0.67 ± 0.82	0.66 ± 0.97	0.66 ± 0.98	0.02	0.01	0.00
Months in treatment, mean ± SD (median; range)^a^	11.13 ± 0.18 (11.07; 10.81–11.50)	12.08 ± 1.72 (12.32; 1.31–14.03)	11.69 ± 0.86 (11.86; 4.34–14.52)	−0.70	−0.61	−0.24
TTSTAND velocity, rises per second, mean ± SD	0.19 ± 0.06	0.19 ± 0.06	0.19 ± 0.06	0.00	0.00	0.00
6MWT distance, m, mean ± SD^b^	313 ± 56	343 ± 34	302 ± 88	−0.54	0.17	−0.67
TTRW velocity, m/s, mean ± SD	1.60 ± 0.36	1.58 ± 0.33	1.63 ± 0.22	0.05	−0.08	−0.13
NSAA total score, n, mean ± SD	18.9 ± 4.1	18.9 ± 4.0	18.9 ± 4.0	0.00	0.00	0.00

Abbreviations: 6MWT = 6-minute walk test; BMI = body mass index; NSAA = North Star Ambulatory Assessment; SMD = standardized mean difference; TTRW = time to run/walk 10 m; TTSTAND = time to stand from supine.

a A single value was missing for the vamorolone 6 mg/kg/d months in treatment calculation.

b 6MWT distance is missing 2 values for vamorolone 6 mg/kg/d, 1 for deflazacort, and 4 for prednisone.

### Efficacy Analyses

**Figure 2:**
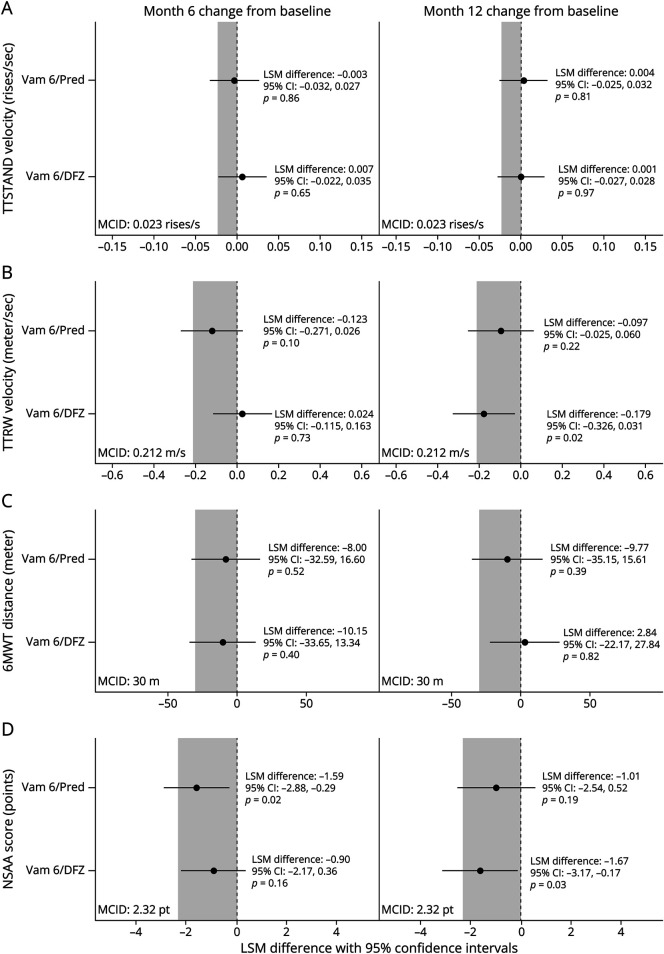
Forest Plots of Treatment Group Differences Group differences are shown in least-squares mean changes from baseline in time to stand from supine velocity (A), time to run/walk 10-m velocity (B), 6-minute walk test distance (C), and North Star Ambulatory Assessment total score (D) at 6 and 12 months of treatment in the VISION-DMD and FOR-DMD trials using weighted data. Circles represent the least squares mean differences and horizontal lines are 95% CIs. Shaded regions represent the literature-defined minimal clinically important difference thresholds*; if the lower bounds of the 95% CIs for the vamorolone comparisons with prednisone or deflazacort fall below the minimal clinically important difference threshold, one cannot reject the null hypothesis of a clinically important group difference. *p* Values were not adjusted for multiple testing. *MCID of TTSTAND velocity, 0.023 rises per second^27^; TTRW velocity, 0.212 m/s^27^; 6MWT distance, 30 m^28^; and NSAA total score, 2.32 points.^29,30^ *p* Values are derived from the MMRM results shown in detail in Table 2. 6MWT = 6-minute walk test; DFZ = deflazacort 0.9 mg/kg/d; LSM = least squares mean; MCID = minimal clinically important difference; NSAA = North Star Ambulatory Assessment; Pred = prednisone 0.75 mg/kg/d; pt = points; TTRW = time to run/walk 10 m; TTSTAND = time to stand from supine; Vam 6 = vamorolone 6 mg/kg/d.

When comparing the LSM change from baseline between treatments in the entropy-balanced data, vamorolone 6 mg/kg/d TTSTAND velocity and 6MWT distance were not statistically different from prednisone and deflazacort at both 6 and 12 months (Table 2; Figure 2). TTRW velocity change from baseline with vamorolone 6 mg/kg/d was not significantly different to prednisone at 6 and 12 months and deflazacort at 6 months but decreased compared with deflazacort at 12 months (LSM difference −0.179 m/s; 95% CI −0.326 to −0.031; *p* = 0.02). NSAA total score changes from baseline were lower with vamorolone 6 mg/kg/d vs prednisone at 6 months (LSM difference −1.59; 95% CI −2.88 to −0.29; *p* = 0.02) and deflazacort at 12 months (LSM difference −1.67; 95% CI −3.17 to −0.17; *p* = 0.03). Notably, the lower bounds of the 95% CIs for vamorolone 6 mg/kg/d comparisons with both prednisone and deflazacort generally overlapped with minimal clinically important difference (MCID) thresholds. Exceptions in these observations were for comparisons with deflazacort regarding TTSTAND velocity, TTRW velocity, and NSAA total score at 6 months and 6MWT distance at 12 months (Figure 2).

**Table 2:** Comparisons of LSM Changes From Baseline Between Treatments at 6 and 12 Months for TTSTAND Velocity, TTRW 10-m Velocity, 6MWT Distance, and NSAA Total Score Performed Using Weighted Data

Outcome	Treatment comparison	6 mo			12 mo		
		n^a^	LSM difference; 95% CI	*p* Value	n^a^	LSM difference; 95% CI	*p* Value
TTSTAND velocity (rises per second)	Vam 6/Pred	27/47	−0.003; −0.032, 0.027	0.86	27/46	0.004; −0.025, 0.032	0.81
	Vam 6/DFZ	27/53	0.007; −0.022, 0.035	0.65	27/51	−0.001; −0.027, 0.028	0.97
TTRW velocity (m/s)	Vam 6/Pred	25/46	−0.123; −0.271, 0.026	0.10	24/46	−0.097; −0.254, 0.060	0.22
	Vam 6/DFZ	25/54	0.024; −0.115, 0.163	0.73	24/51	−0.179; −0.326, −0.031	0.02
6MWT distance (m)	Vam 6/Pred	20/45	−8.00; −32.59, 16.60	0.52	19/46	−9.77; −35.15, 15.61	0.45
	Vam 6/DFZ	20/51	−10.15; −33.65, 13.34	0.39	19/49	2.84; −22.17, 27.84	0.82
NSAA score (points)	Vam 6/Pred	26/48	−1.59; −2.88, −0.29	0.02	24/47	−1.01; −2.54, 0.52	0.19
	Vam 6/DFZ	26/53	−0.90; −2.17, 0.36	0.16	24/50	−1.67; −3.17, −0.17	0.03

Abbreviations: 6MWT = 6-minute walk test; DFZ = deflazacort 0.9 mg/kg/d; LSM = least squares mean; NSAA = North Star Ambulatory Assessment; Pred = prednisone 0.75 mg/kg/d; TTRW = time to run/walk 10 m; TTSTAND = time to stand from supine; Vam 6 = vamorolone 6 mg/kg/d.

*p* Values were not adjusted for multiple testing.

a Raw numbers of boys who had outcome data available at the given time point before weighting. Missing data were accommodated using the missing-at-random assumption.

### Anthropometric Analyses

**Figure 3:**
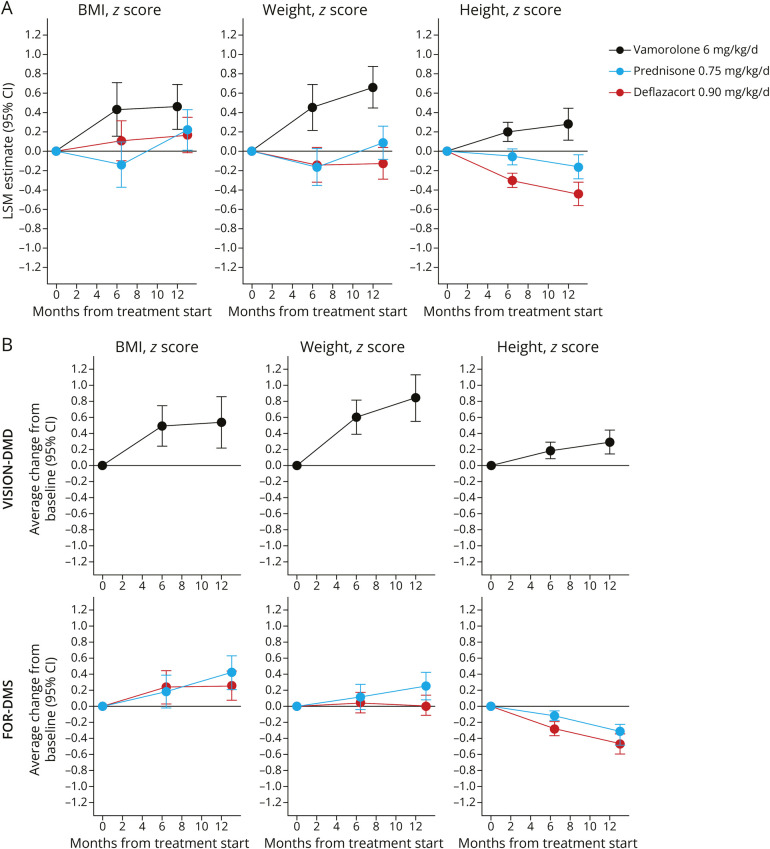
Changes From Baseline in BMI, Weight, and Height *Z*-Scores (A) LSM estimates with 95% CI, for comparisons between vamorolone 6.0 mg/kg/d, prednisone 0.75 mg/kg/d, and deflazacort 0.90 mg/kg/d. LSM differences are derived from a weighted MMRM with baseline response, month (weeks 24 and 48 for VISION-DMD and months 6 and 12 for FOR-DMD), treatment, and a treatment-by-visit interaction.* (B) Arithmetic mean changes from baseline with 95% CI. CIs are derived using a t-distribution with n - 1 degrees of freedom where n denotes the number of individuals per treatment and visit. The estimated LSMs in panel A are adjusted for baseline, whereas panel B does not contain such an adjustment. *In panel A, the FOR-DMD population has been reweighted so that the baseline characteristics in key variables match those in VISION-DMD. BMI = body mass index; LSM = least squares mean; MMRM = mixed model for repeated measures; SE = standard error.

LSM estimates of BMI *Z*-scores increased in all treatment groups at 12 months, with a decrease in BMI observed for prednisone at 6 months (prednisone vs vamorolone 6 mg/kg/d at 6 months, BMI *Z*-score LSM difference 0.57; 95% CI 0.24–0.90; *p* = 0.009) and a smaller increase for deflazacort compared with vamorolone at 12 months (LSM difference 0.29; 95% CI 0.03–0.56; *p* = 0.03) (Table 3; Figure 3A). Weight *Z*-score increases were greatest in the vamorolone 6 mg/kg/d group compared with prednisone and deflazacort at 6 and 12 months (all comparisons *p* < 0.0001) (Table 3; Figure 3A). Slowdown in growth, demonstrated by increasingly negative changes in height *Z*-scores, occurred in the prednisone- and deflazacort-treated but not the vamorolone-treated groups at 6 and 12 months (all comparisons *p* < 0.0001; Table 3; Figure 3A). For comparison, the arithmetic mean anthropometric *Z*-scores from data that have not been reweighted to match baseline characteristics are shown in Figure 3B.

**Table 3:** Comparisons of LSM Changes From Baseline Between Treatments at 6 and 12 Months for Anthropometric Outcomes Performed Using Weighted Data

Outcome	Treatment comparison	6 mo			12 mo		
		n^a^	LSM difference; 95% CI	*p* Value	n^a^	LSM difference; 95% CI	*p* Value
BMI *Z*-score	Vam 6/Pred	26/48	0.57; 0.24, 0.90	0.009	26/48	0.24; −0.04, 0.52	0.09
	Vam 6/DFZ	26/54	0.32; 0.00, 0.64	0.05	26/51	0.29; 0.03, 0.56	0.03
Weight *Z*-score	Vam 6/Pred	27/48	0.61; 0.34, 0.89	<0.0001	26/48	0.58; 0.33, 0.82	<0.0001
	Vam 6/DFZ	27/54	0.60; 0.33, 0.87	<0.0001	26/51	0.78; 0.54, 1.02	<0.0001
Height *Z*-score	Vam 6/Pred	26/48	0.25; 0.14, 0.36	<0.0001	26/48	0.44; 0.25, 0.64	<0.0001
	Vam 6/DFZ	26/51	0.50; 0.39, 0.61	<0.0001	26/51	0.72; 0.53, 0.91	<0.0001

Abbreviations: BMI = body mass index; DFZ = deflazacort 0.9 mg/kg/d; Pred = prednisone 0.75 mg/kg/d; vam 6 = vamorolone 6 mg/kg/d.

*p* Values were not adjusted for multiple testing.

a Raw numbers of boys who had outcome data available at the given time point before weighting. Missing data were accommodated using the missing-at-random assumption.

### Daily Vamorolone 2 mg/kg/d vs Intermittent Prednisone 0.75 mg/kg 10 Days On/10 Days Off

**Figure 4:**
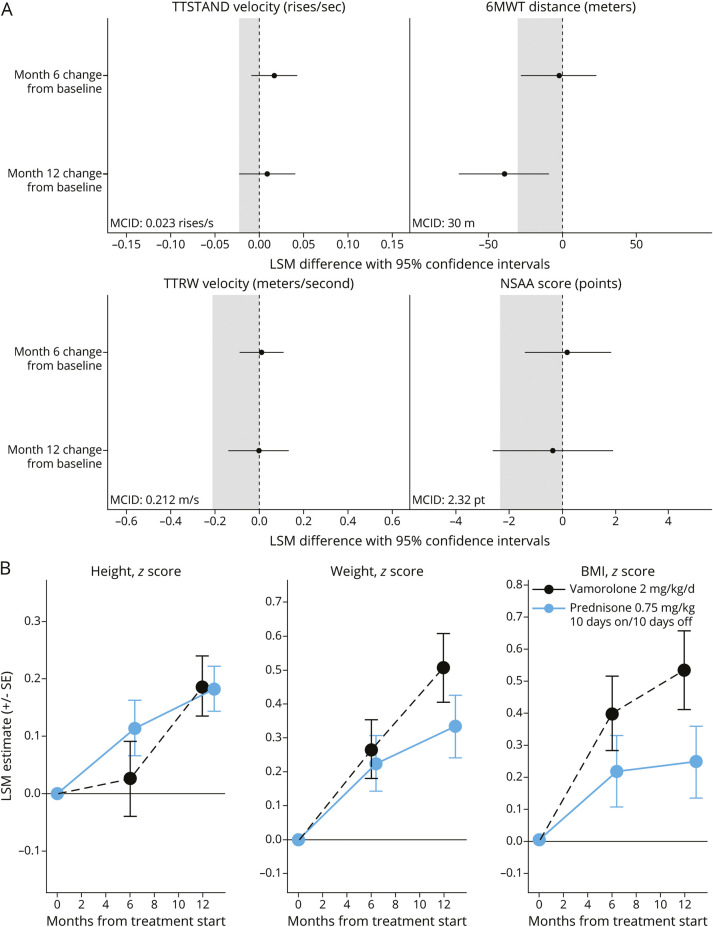
Vamorolone 2 mg/kg/d vs Intermittent Prednisone 0.75 mg/kg/d (10 Days On/10 Days Off) (A) Forest plots of differences between vamorolone 2 mg/kg/d and intermittent prednisone 0.75 mg/kg/d in least-squares mean changes from baseline. Group differences shown in time to stand from supine velocity, 6-minute walk test distance, time to run/walk 10-m velocity, and North Star Ambulatory Assessment total score at 6 and 12 months of treatment in the VISION-DMD and FOR-DMD trials using weighted data. Circles represent the least squares mean differences, and horizontal lines are 95% CIs. Shaded regions represent the literature-defined minimal clinically important difference thresholds*; if the lower bounds of the 95% CIs for the vamorolone comparisons with prednisone fall below the minimal clinically important difference threshold, one cannot reject the null hypothesis of a clinically important group difference. *p* Values were not adjusted for multiple testing. (B) Changes in anthropometric values, height, weight, and BMI *Z*-scores, from baseline. *MCID of TTSTAND velocity, 0.023 rises per second^27^; TTRW velocity, 0.212 m/s^27^; 6MWT distance, 30 m^28^; and NSAA total score, 2.32 points.^29,30^ 6MWT = 6-minute walk test; DFZ = deflazacort 0.9 mg/kg/d; LSM = least squares mean; MCID = minimal clinically important difference; NSAA = North Star Ambulatory Assessment; Pred = prednisone 0.75 mg/kg/d (10 days on/10 days off); pt = points; SE = standard error; TTRW = time to run/walk 10 m; TTSTAND = time to stand from supine; Vam 2 = vamorolone 2 mg/kg/d.

Descriptive statistics and SMD for vamorolone 2 mg/kg/d and intermittent prednisone entropy-balanced and weighted data achieved effective balancing between cohorts (eTable 1; eFigures 3 and 4). At 6 and 12 months of treatment, observed mean changes from baseline in efficacy outcomes were generally similar between the treatment groups, with only the 95% CI for NSAA score crossing the MCID threshold, whereas LSM 6MWT distance was shorter with vamorolone 2 mg/kg/d vs intermittent prednisone 0.75 mg/kg at 12 months (LSM difference −39.56; 95% CI −69.92 to −9.19; *p* = 0.01 Figure 4). Observed mean anthropometric outcomes were similar between vamorolone 2 mg/kg/d and intermittent prednisone, with changes in weight and BMI tending to be higher with vamorolone 2 mg/kg/d at 12 months (Figure 4).

### Classification of Evidence

This Class III study did not definitively identify differences in efficacy between vamorolone and classic corticosteroids but found that vamorolone protects linear growth in boys with DMD.

## Discussion

Using entropy balanced weighting and MMRM, the efficacy of vamorolone 6 mg/kg/d was compared with daily prednisone and deflazacort for corticosteroid-naïve boys with DMD using data from 2 randomized trials, VISION-DMD and FOR-DMD. The VISION-DMD trial was designed after FOR-DMD with consideration for potential cross-trial analyses enabled by harmonized efficacy measures. Of note, in all the studies discussed, TTSTAND and TTRW outcomes were converted to velocities using the measurement reciprocal (meters/second for TTRW), with increased velocities indicative of improved muscle function. Converting these outcomes to velocities reduces outlier effect, preserves data for participants that lose the ability to stand, and provides greater resolution of changes in measures at clinically meaningful levels. Furthermore, a recent assessment of outcome reliability by age groups across VISION-DMD and FOR-DMD showed generally good reliability and agreement in measures used across the 2 trials.^31^ In both VISION-DMD and FOR-DMD, vamorolone, prednisone, and deflazacort improved motor function from baseline. At 48 weeks in VISION-DMD, all motor outcome measures for vamorolone 6 mg/kg/d were stable or showed continued improvements.^20^ FOR-DMD did not include a placebo group, but both daily prednisone and deflazacort groups showed similar improvements in all motor outcome measures to 6 months; these measures were then stable or improved further to 12 months.^13^

The current cross-study indirect comparison using VISION-DMD and FOR-DMD weighted data found that vamorolone treatment at the recommended starting dosage of 6 mg/kg/d showed mixed efficacy responses vs the classic corticosteroids. Despite similar study designs in VISION-DMD and FOR-DMD, interpretation of the comparative efficacy results presented here is limited by the relatively small sample sizes and wide CIs for group differences in LSMs. Although comparisons for some efficacy measures between vamorolone 6 mg/kg/d, prednisone 0.75 mg/kg/d, and deflazacort 0.9 mg/kg/d yielded similar LSMs, the 95% CIs for most of these group differences crossed MCID thresholds. The relative efficacy of vamorolone 6 mg/kg/d and deflazacort 0.9 mg/kg/d, which has yet to be directly studied, was, therefore, not fully clarified by the current analysis. For prednisone, previously, vamorolone 6 mg/kg/d and 2 mg/kg/d were compared directly during the initial 24-week period of the randomized double-blind VISION-DMD study as an exploratory analysis. This previous within-study comparison at 6 months reported that vamorolone 6 mg/kg/d and prednisone 0.75 mg/kg/d achieved similar relative efficacy for all 5 motor function measures tested, 4 of which are included in the current analysis (TTSTAND velocity, TTRW velocity, 6MWT distance, and NSAA score), with the caveats of relatively small sample sizes, and VISION-DMD was not designed as a noninferiority study.^20,21^

Anthropometric measurements, specifically height, weight, and BMI *Z*-scores, were used to track corticosteroid-associated side effects in both the VISION-DMD and FOR-DMD trials. *Z*-scores indicate the number of standard deviations between a measure and the healthy population mean value, accounting for age and sex; the more the absolute *Z*-score deviates from 0, the greater the difference in height/weight/BMI between the boy with DMD and the average reference “healthy” boy.^32^ A change in height *Z*-score relative to the study baseline of 0 suggests that a patient is remaining on the same height trajectory, a decrease over time suggests a slowdown of growth, and a consistent rapid increase after a period of growth retardation could suggest catch up growth.^32,33^ For anthropometric effects, vamorolone was not associated with slowdown in growth, a key side effect of classic daily corticosteroids also apparent in the data presented here.^34^ The LSM estimates of *Z*-scores after entropy balancing presented here are derived from a weighted regression model, hence, differences between the raw and balanced data. As such, these LSM estimates should only be used for the comparison of treatment effects rather than interpreting absolute values.

Previous comparisons with “healthy” reference populations have shown that boys with DMD have both cross-sectionally lower height *Z*-scores after 2 years of age relative to reference populations and experience slowdown in growth over time.^35^ These observations result from an inherent short stature, even in corticosteroid-naïve individuals.^35–37^ This is in addition to other clinical factors associated with DMD that can adversely affect the evolution of height *Z*-scores, including progressive scoliosis, delayed puberty, muscle atrophy, and corticosteroid-associated growth failure.^38,39^ In the current cross-study weighted analysis, a slowdown in growth was observed in both prednisone- and deflazacort-treated boys but not with vamorolone, supporting previous results.^20,21^ In contrast to the classic corticosteroids, the change from baseline in average height *Z*-score of boys treated with vamorolone increased over the 48-week time period of VISION-DMD (i.e., positive change from baseline); however, the absolute *Z*-scores remained negative during the study period.^20^ Of note, all weighted groups had height *Z*-scores of −1.04 at baseline, indicative of delayed or reduced growth, and by week 48, vamorolone-treated patients had made a small *Z*-score LSM-estimate gain of approximately 0.3, whereas height *Z*-score LSM estimates had further decreased by approximately 0.2 and 0.5 for prednisone and deflazacort, respectively. These findings are further supported by the observation from the VISION-DMD trial in which 24-week prednisone treatment induced a slowdown in growth that was reversed through catch-up growth after switching to vamorolone after a 4-week transition period.^20,21^ In the same study, at 24 weeks, the height *Z*-score change from baseline in the vamorolone and placebo groups were similar, suggesting vamorolone had no negative effect on growth.^20,21^ In both instances, the overall trend is for vamorolone to maintain growth trajectories rather than cause growth retardation. For context, typical annual height *Z*-score variability among healthy children aged 4–7 years is ±0.1 standard deviations,^40^ and a change of >0.25 is rarely seen in longitudinal growth studies.^33^ The trend for vamorolone to enable patients to maintain their growth was also seen in the indirect post-hoc comparison between vamorolone 2 and 6 mg/kg during the 30-month open-label, nonrandomized phase 2a trial extension period and the CINRG DNHS, in which upward growth trajectories occurred in the vamorolone-treated boys, in contrast to the slowdown in growth observed in the classic corticosteroid–treated CINRG DNHS external control dataset.^22^ Taken together, vamorolone appears to dissociate muscle-strength efficacy from the slowdown in growth associated with classic corticosteroids.

In addition to slower growth, classic corticosteroids are also associated with weight gain. All treatments in the current analysis demonstrated increases in BMI *Z*-scores; however, the relationship between treatments and weight changes varied, with vamorolone 6 mg/kg/d associated with the greatest weight *Z*-score LSM estimate increases, which cannot be interpreted independently from the change in height. In this context, BMI is arguably a better reflection of weight status, because this measure considers that changes in weight are scaled to changes in height. To this end, we found that the increase in the BMI *Z*-score from baseline was greater on vamorolone than prednisone at 6 but not at 12 months. In contrast, the change in BMI *Z*-score from baseline on vamorolone was greater than that on deflazacort at 12 months. Overall, anthropometric *Z*-scores associated with the classic corticosteroids show that deflazacort had greater effect on slowing growth and prednisone had greater effect on weight gain relative to each other. Because of the increases in BMI across treatments, a nutritional plan should be implemented with suitable weight-gain prevention strategies for all 3 treatments as per current guidance.^11^

The comparison of vamorolone 2 mg/kg/d with intermittent (10 days on/10 days off) prednisone 0.75 mg/kg showed generally similar effects on motor function outcomes, except for reduced improvement of 6MWT distance with vamorolone 2 mg/kg/d at 12 months. Vamorolone 2 mg/kg/d previously demonstrated suboptimal efficacy in other studies.^20,21^ Clinicians and families may, nevertheless, consider down-titration from vamorolone 6 mg/kg to 4 mg/kg/d or 2 mg/kg/d based on individual tolerability, as per prescribing information. However, it should be recognized that the highest tolerated and indicated dosage of vamorolone should be maintained to optimize efficacy.^14^

A direct comparison of safety was not possible in the current analysis due to differences in adverse event reporting that can be seen in the higher number of events associated with prednisone in VISION-DMD vs the FOR-DMD prednisone data.^13,21^ Reasons for this discrepancy include a higher frequency of on-site visits and laboratory assessments in VISION-DMD, which was an industry-sponsored and managed placebo-controlled drug registration clinical trial with extensive pharmacovigilance. Of note, VISION DMD provided safety data that show that vamorolone causes adrenal suppression in a dosage-dependent fashion, similar to classic corticosteroids, leading to the recommendation that all patients receiving vamorolone, regardless of dosage, should receive “steroid stress dosing” with prednisone or hydrocortisone (but not vamorolone) at times of surgery, injury, or moderate/severe illnesses to prevent adrenal insufficiency crises analogous to the recommendations for classic corticosteroid therapy.^41,42^ The VISION-DMD study also suggested a descriptive reduction in behavioral side effects with vamorolone compared with prednisone during the first 24 weeks of treatment, and further analyses are ongoing to explore this.^20,21^

Because of the cross-study nature of the comparisons presented, interpretation and extrapolation of the data are limited. Overall, VISION-DMD and FOR-DMD had similar designs; however, the most prominent differences included the eligibility of boys 4 to <8 years of age in FOR-DMD vs 4 to <7 years of age in VISION-DMD, the requirement for boys to be able to complete TTSTAND in <10 seconds in VISION-DMD, and the upper weight limit of ≤39.9 kg at screening in VISION-DMD. Nevertheless, all these aspects were accounted for through the eligibility criteria used to derive the analysis cohorts. The entropy balancing method resulted in effective balancing of treatment groups. In contrast to propensity score matching, entropy balancing enabled the inclusion of all eligible patients to ensure the broadest comparison across the trial populations. Despite this, there were some differences between baseline characteristics, particularly in 6MWT distance, which was higher for prednisone than for the other groups, TTRW velocity, which was lower in the prednisone groups than in the other groups, and NSAA, which was lower in the vamorolone groups. In addition to the statistical limitations described earlier regarding small sample sizes and inconclusive results concerning the efficacy comparisons, differences in assessment schedules should also be considered. The VISION-DMD trial assessed participants at the 12-, 24- and 48-week time points, whereas comparable measurements in FOR-DMD were at 6 and 12 months (26 and 52 weeks, respectively). This difference in assessment schedule meant that average time in treatment was marginally longer for the classic corticosteroids vs vamorolone. In addition, FOR-DMD permitted dosage reduction in response to adverse events whereas VISION-DMD did not. This means that the actual received dosages may have decreased from the starting dosage for the prednisone and deflazacort groups. Finally, our results provide insights in young, baseline-corticosteroid-naïve boys with DMD, but the long-term effect of treatment choice remains to be determined in older patients as DMD progresses. Moving forward, a greater number of patients will need to be followed to further elucidate the differences between these treatments, especially in clinical practice where dosage reductions in response to adverse effects may determine patient outcomes and in an aging population where additional health-related considerations may occur.

This cross-study entropy-balanced weighted analysis was unable to definitively identify differences in efficacy between daily vamorolone 6 mg/kg/d and daily classic corticosteroids at recommended starting dosages for DMD over 1 year. Comparisons of anthropometric data between VISION-DMD and FOR-DMD expand on previous observations that vamorolone may dissociate efficacy from corticosteroid-associated slowdown in growth observed in prednisone- and deflazacort-treated boys. All 3 treatments were associated with increases in weight and BMI *Z*-scores. The linear growth-trajectory-protective feature of vamorolone, together with descriptive reductions in behavioral problems and improved bone biomarker profile as seen in the original VISION-DMD analysis,^20^ may reduce the real-world frequency of dosage reductions and treatment cessation vs prednisone and deflazacort; additional studies are needed to test that hypothesis. Overall, vamorolone is indicated for patients with DMD aged 4 years and older (European Union and United Kingdom) and in patients aged 2 years and older (United States), and in the current analysis, we have shown evidence of a growth protective effect in contrast to the classic corticosteroids.

## Glossary

6MWT: 6-minute walk test
ATT: average treatment effect in the treated
BMI: body mass index
CINRG DNHS: CINRG Duchenne Natural History Study
DMD: Duchenne muscular dystrophy
FOR-DMD: Finding the Optimum Steroid Regimen for DMD
LSM: least squares mean
MCID: minimal clinically important difference
MMRM: mixed model for repeated measures
NSAA: North Star Ambulatory Assessment
SMD: standardized mean difference
TTRW: time to run/walk
TTSTAND: time to stand from supine
